# Post-translational modifications of HTLV-1 Tax carboxy-terminal domain: role in cellular transformation

**DOI:** 10.1186/1742-4690-11-S1-O41

**Published:** 2014-01-07

**Authors:** Julie Lodewick, Anne Sophie Rinaldi, Françoise Bex

**Affiliations:** 1 Institute for Microbiological Research J-M Wiame, Université Libre de Bruxelles, Belgium

## 

Transformation of T lymphocytes by HTLV-1 is linked to the capacity of its oncoprotein Tax to interfere with cellular regulatory pathways, including control of cell cycle progression and inactivation of tumor suppressor p53. Tax is modified by a hierarchical sequence of post-translational modifications that controls its intracellular localization and functions via crosstalk. Among these modifications, phosphorylation and acetylation occur at S336 and K346, respectively, in the carboxy-terminal domain of Tax, a domain involved in Tax transforming activity. We determined that the acetylation deficient K346R mutant had a markedly reduced capacity to transform Rat-1 fibroblast, as compared to the acetyl-mimetic K346Q mutant, which transformed Rat-1 cells as wild type Tax. This property correlated with the reduced capacity of mutant K346R to bypass inhibition of CDK4/cyclin D3 complexes by p21, resulting in its reduced capacity to stimulate pRb kinase activity of CDK4. Thus, acetylation at lysine residue 346 by the acetyltransferase p300 likely directly participates in the Tax transforming activity. We also determined that phosphorylation of Tax at the S336P motif involved the proline-directed serine/threonine kinase HIPK2. HIPK2 interacts with Tax and is recruited in the Tax nuclear bodies. Furthermore, the kinase activity of HIPK2 is required for efficient functional inactivation of p53 by Tax. These results indicate that modifications of the Tax carboxy-terminal domain control at least two critical functions in Tax transforming activities: stimulation of cell cycle progression through G1/S via activation of CDK4 kinase activity and functional inactivation of p53 via recruitment of HIPK2 kinase in Tax nuclear bodies.

